# Diffuse panbronchiolitis as a rare complication of thymectomy and radiation therapy in a patient with thymoma: a case report

**DOI:** 10.3389/fonc.2025.1496693

**Published:** 2025-01-30

**Authors:** Ye Lu, Qi Qi, Dan Qu, Yu Chen

**Affiliations:** Department of Pulmonary and Critical Care Medicine, Shengjing Hospital of China Medical University, Shenyang, China

**Keywords:** diffuse panbronchiolitis, thymoma, cellular immunodeficiency, radiation, case report

## Abstract

**Background:**

Diffuse panbronchiolitis (DPB) is an uncommon respiratory disorder characterized by the presence of respiratory bronchiolitis and persistent inflammation in adjacent tissues, which can be effectively treated with early diagnosis and intervention. DPB is a rare complication associated with thymoma that remains poorly understood, especially when it occurs in conjunction with acquired cellular immune deficiency.

**Case presentation:**

We present a case of DPB in a patient with thymoma following thymectomy and radiation therapy. A 47-year-old Chinese man underwent thymectomy due to the presence of a mediastinal mass, and pathological examination confirmed a type B2 thymoma. He also underwent 25 sessions of radiation therapy. The patient’s respiratory symptoms, including cough, expectoration, and shortness of breath, worsened significantly after radiation treatment. Immune dysfunction, marked by CD4+ T cell immunodeficiency with normal immunoglobulin levels, was observed. Chest computed tomography revealed diffuse nodules with tree-in-bud signs and new consolidation within the irradiated area, leading to a diagnosis of combined DPB and radiation pneumonitis. The patient’s symptoms and lung imaging findings significantly improved after the initiation of low-dose oral azithromycin for DPB and low-dose glucocorticoid therapy for radiation pneumonitis.

**Conclusions:**

Clinicians should consider DPB in patients with thymoma and cellular immunodeficiency. Both thymectomy and radiation therapy can contribute to the development of DPB. Early treatment with macrolides can improve patient prognosis.

## 1 Introduction

Thymomas are common mediastinal masses in adults (1) and are associated with various paraneoplastic autoimmune syndromes, including myasthenia gravis (2), pure red cell aplasia (3), and Addison’s disease (4). Diffuse panbronchiolitis (DPB) is a rare respiratory disease characterized by respiratory bronchiolitis and chronic inflammation of the surrounding tissues (5). The lesions associated with this condition affect the entire layer of the respiratory bronchiole wall, leading to its classification as “panbronchiolitis” (6). DPB is rarely documented as a paraneoplastic autoimmune syndrome in patients with thymomas. The association between DPB and thymoma has not garnered substantial attention from clinicians, highlighting the need for further investigation of its clinical characteristics and correlation with immune deficiency. DPB can be successfully treated if diagnosed early but may progress to irreversible bronchiectasis in advanced stages (7). Therefore, early diagnosis of patients with thymomas is crucial.

Radiotherapy is a key component of the multimodal approach for the management of various cancers, including thymomas (8, 9). With continuous advancements in radiation technology, the incidence of radiation-related adverse effects has been significantly reduced; however, radiation-induced side effects such as radiation pneumonitis still occur (9). Radiotherapy can promote or exacerbate immune dysfunction (10). Although the relationship between radiation therapy and cellular immune dysfunction in thymomas has been reported in only a few cases (11–13), the close association between thymomas and autoimmunity requires that greater clinical attention should be paid to this phenomenon.

This study presents a unique case of a patient with thymoma who experienced a decrease in CD4+ T lymphocytes following thymectomy and radiation therapy despite maintaining normal immunoglobulin levels. Notably, the patient’s chest computed tomography (CT) revealed multiple diffuse nodules and tree-in-bud signs resembling DPB, along with new infiltrations and consolidations in the lungs. To the best of our knowledge, no previous study has reported similar findings. This case report provides a comprehensive diagnostic analysis of DPB and radiation pneumonitis in a patient with thymoma after radiation therapy. Remarkably, the use of macrolide antibiotics led to a significant improvement in DPB, whereas glucocorticoid therapy resulted in substantial resolution of radiation pneumonitis. The effective identification and management of DPB in patients with thymoma could offer valuable insights for clinical practitioners.

## 2 Case description and diagnostic assessment

**Figure 1:**
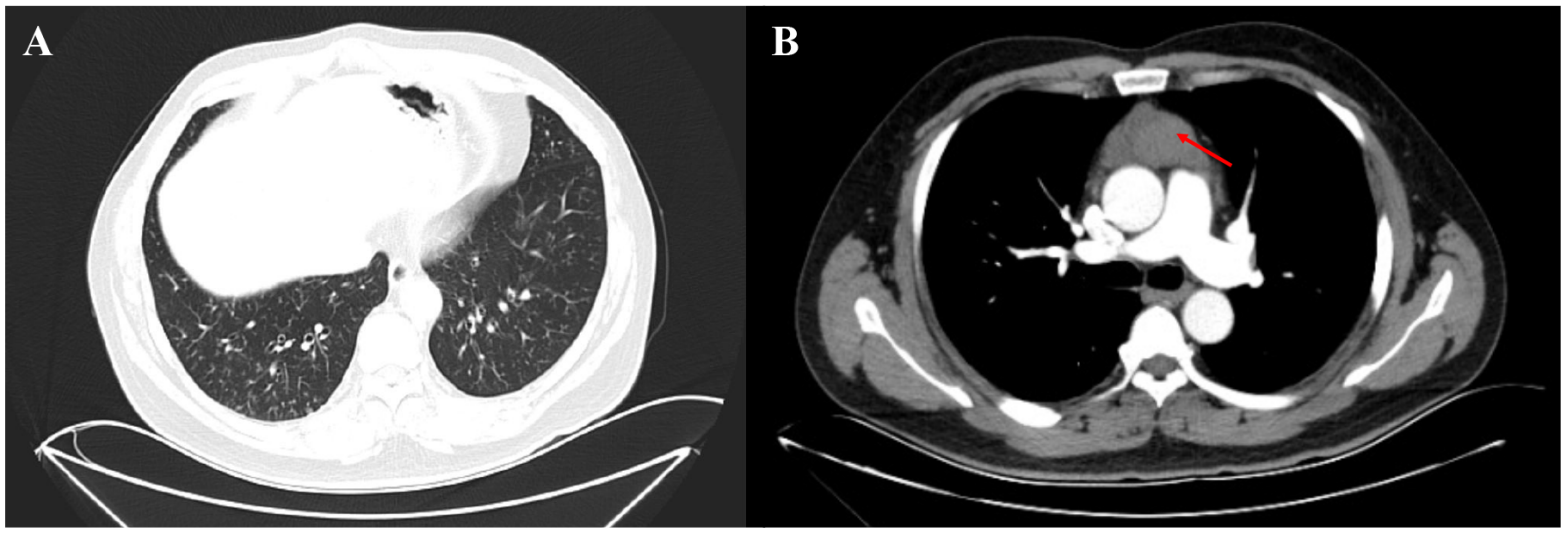
Enhanced chest CT of the patient before thymectomy. **(A)** Small nodules were distributed throughout both lungs, with a predominant presence in the lower lobes (pulmonary window). **(B)** The thymus region (red arrow) located in the anterior mediastinum exhibited increased density and heterogeneity, with the enhanced scan revealing a slight enhancement.

**Figure 2:**
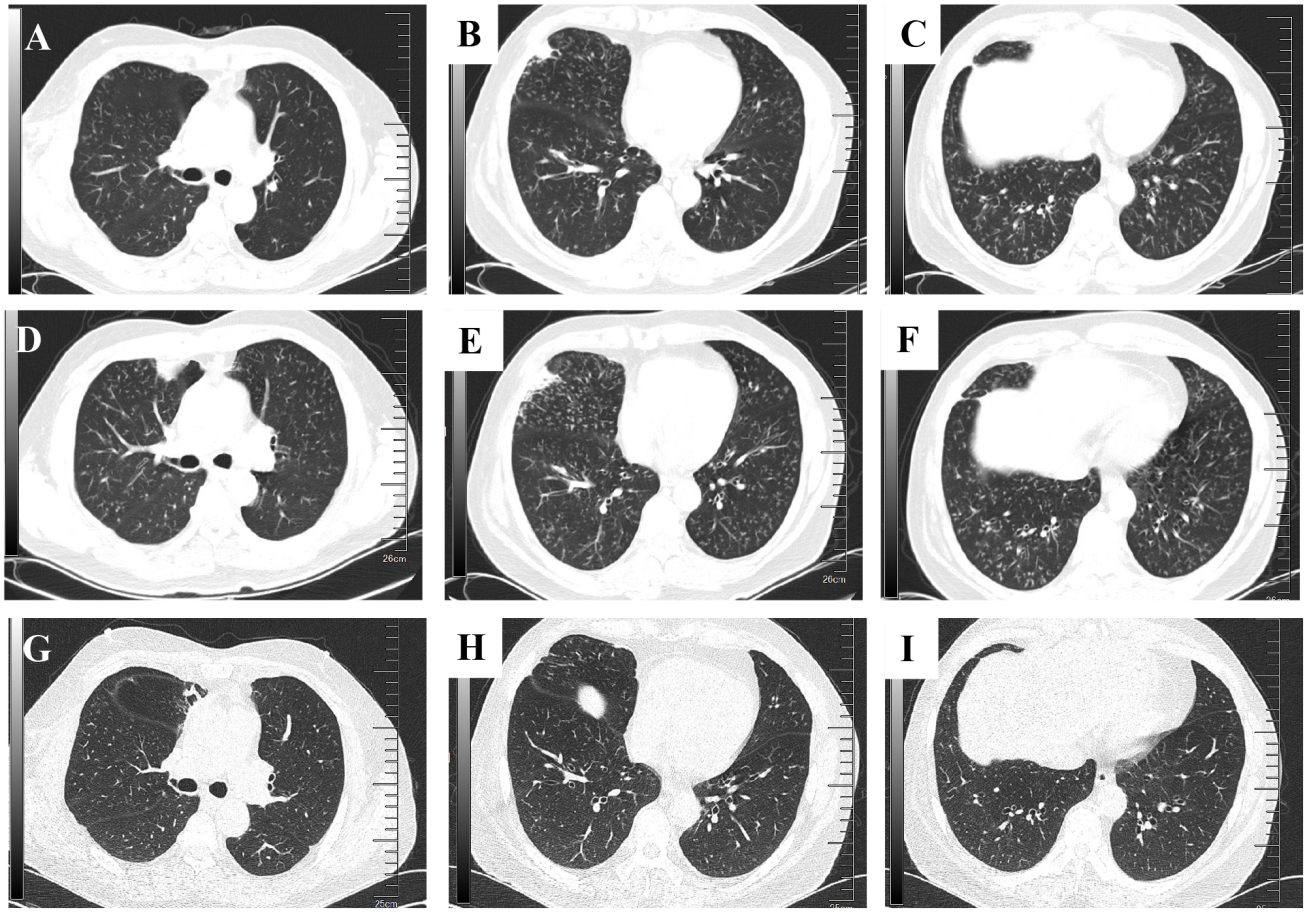
Chest CT scans of the patient. **(A–C)**: The chest CT (2021-1-28) during the period of thymectomy and radiation showed that the distribution of small nodules in both lungs increased significantly, accompanied by changes in tree bud signs. Additionally, an emerging consolidation opacity in the middle lobe of the right lung. **(D–F)**: The chest CT (2021-04-03) after 25 thymectomies and radiation showed that the number of nodules and the presence of tree bud signs in both lungs exhibited a greater degree of progression. The area of consolidation opacity in the middle lobe of the right lung enlarged, while a novel consolidation opacity emerged in the upper lobe of the right lung. **(G–I)**: After treatment with azithromycin and steroids for three months, the chest CT showed that the small nodules were reduced, and the consolidation of the upper lobe and middle lobe of the right lung was absorbed.

A 47-year-old Chinese man presented to the thoracic surgery department of our hospital on October 24, 2020, with recurrent cough, expectoration, and shortness of breath. Chest-enhanced CT revealed a mediastinal mass and small scattered nodules in the lungs (**Figure 1**). The patient refused preoperative positron emission tomography (PET)-CT evaluation. The patient underwent total thymectomy (R0-resection), and pathological examination confirmed a type B2 thymoma. The Masaoka–Koga stage was IIA, and the tumor–node–metastasis stage was T1N0M0 (stage I). Comprehensive consideration of the Masaoka–Koga stage, WHO subtype, and the patient’s wish, the patient received 25 sessions of radiation therapy (total dose 50 Gy) from December 17, 2020, to February 3, 2021. During radiation therapy, his cough, expectoration, and shortness of breath gradually worsened, and chest CT revealed new consolidation in the middle lobe of the right lung and a significant increase in small pulmonary nodules compared to the pre-radiation period (**Figures 2A–C**). The patient underwent a PET-CT examination at another hospital, which showed that multiple miliary nodules in both lungs were non-hypermetabolic, whereas the new consolidation in the middle lobe of the right lung was hypermetabolic (maximum SUV: 3.3). On April 3, 2021, the patient visited our respiratory department because of worsening respiratory symptoms, including cough, increased yellow sputum, and shortness of breath. Lung function tests indicated obstructive pulmonary ventilation dysfunction, with a forced expiratory volume in one second/forced vital capacity ratio (FEV_1_/FVC) of 0.69, a forced expiratory volume in one second of the predicted value (FEV_1_/pre) of 79%, and normal pulmonary diffuse capacity for carbon monoxide (DLCO). A CT scan (**Figures 2D–F**) revealed multiple diffuse small nodules in both lungs, accompanied by tree-in-bud signs. Additionally, a new consolidation was observed in the upper lobe of the right lung, along with an enlargement of the irregular consolidation in the middle lobe of the right lung. The patient had a history of chronic paranasal sinusitis. He denied a history of smoking, dust inhalation, long-term use of specific medications, family history of lung diseases, or pet ownership. Upon admission, physical examination revealed coarse crackles in both lungs.

The primary laboratory results are listed in **Supplementary Table S1**. Arterial blood gases on room air showed a partial oxygen pressure of 65 mmHg. White blood cell count, neutrophil percentage, and C-reactive protein levels were normal. Multiple sputum cultures yielded negative results. The patient underwent bronchoscopy and bronchoalveolar lavage. The culture of bronchoalveolar lavage fluid (BALF) was negative. However, the identification of a high percentage of neutrophils in the differential cell count of BALF, along with the respiratory symptoms of increased yellow sputum production, suggested a possible respiratory tract infection. Consequently, the patient was treated with piperacillin sodium and tazobactam (4.5 g, three times a day, intravenous infusion) for 13 days. After antibiotic treatment, the yellow sputum improved, but the cough and shortness of breath persisted.

Immune function tests showed a deficiency in the CD4+ T lymphocyte count (253/μL) with normal immunoglobulin levels. Complement 3 and 4 levels were also within normal limits. The antinuclear antibody titer was 1:80, and the antinuclear antibody spectrum was negative. Although myeloperoxidase anti-neutrophil cytoplasmic antibodies (ANCA) were positive, perinuclear ANCA, proteinase 3 ANCA, and cytoplasmic ANCA were negative. Hepatic and renal function tests, as well as routine urine tests, revealed no abnormalities. Therefore, the available evidence was inadequate to support a diagnosis of systemic vasculitis.

Given the presence of multiple small nodules with tree-in-bud signs, the possibility of miliary tuberculosis was considered. Multiple smears and PCR tests for tuberculosis in the sputum and BALF, along with the T-spot test, showed negative results, leading to the exclusion of tuberculosis.

Small nodules in both lungs were not subjected to histopathological evaluation for several reasons. First, PET-CT revealed non-hypermetabolic nodules, further ruling out the possibility of pulmonary metastases. Second, bronchoscopy did not identify any abnormal alterations in the tracheal and bronchial mucosa. Furthermore, the patient’s worsening respiratory symptoms precluded his ability to tolerate percutaneous lung biopsy. However, the final diagnosis of DPB was confirmed based on the diagnostic criteria established by a working group of the Ministry of Health and Welfare of Japan (14), which include respiratory symptoms, such as persistent cough, sputum, dyspnea, chronic paranasal sinusitis, diffuse small nodules on chest CT, FEV_1_/FVC < 70%, and PaO_2_ < 80 mmHg. Nevertheless, the emerging consolidation observed on chest CT cannot be fully explained by DPB alone. Although PET-CT indicated hypermetabolic consolidation in the right lung, the swift development of consolidation opacity was inconsistent with the typical growth patterns of a malignant tumor. It is important to note that non-malignant lesions, such as inflammation, can also manifest as hypermetabolism. The newly observed consolidation was located near the radiation site, lacked evidence of pathogenic infection, and responded poorly to anti-infection treatment, suggesting that radiation pneumonitis could not be ruled out. Given the worsening respiratory symptoms, early initiation of effective treatment was necessary. The patient was treated with 0.25 mg of oral azithromycin daily for DPB and 40 mg of methylprednisolone by intravenous infusion for radiation pneumonitis. The symptoms of cough, expectoration, and shortness of breath improved significantly. The patient was discharged with instructions to continue a regimen of low-dose azithromycin and 30 mg prednisolone daily, with the prednisolone dosage gradually reduced by 5 mg every 2 weeks.

**Figure 3:**
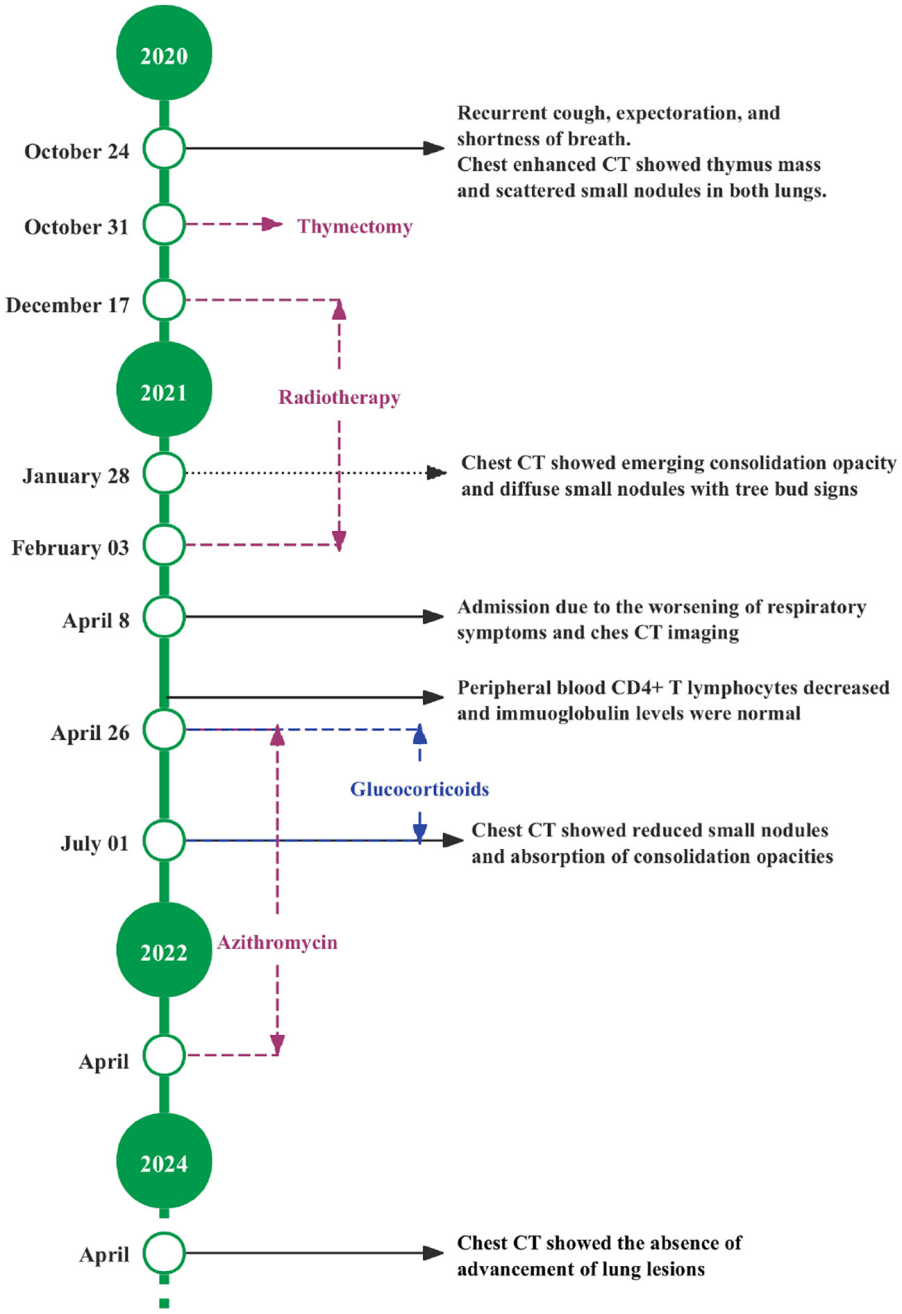
Timeline of the patient’s treatment.

At the follow-up on July 1, 2021, repeat chest CT showed resolution of consolidations in the right middle and upper lobes, a decrease in the number of small nodules, and the presence of improved tree-in-bud signs in both lungs (**Figures 2G–I**). Given the stabilization of respiratory symptoms and the absence of deterioration on lung imaging, azithromycin therapy was discontinued after 2 years. During the latest follow-up on April 22, 2024, the patient’s respiratory symptoms remained stable, and chest imaging demonstrated no progression. The details of the diagnosis and treatment are shown in **Figure 3**.

## 3 Discussion

This study presents the case of a patient with thymoma who exhibited CD4 + T-cell immunodeficiency following thymectomy and radiation. Additionally, the patient’s lungs showed diffuse nodules with tree-in-bud signs and emerging consolidation within the radiation field, which were determined to be a combination of DPB and radiation pneumonitis. Subsequently, the patient’s symptoms and lung imaging findings significantly improved with low-dose azithromycin therapy for DPB and low-dose glucocorticoid therapy for radiation pneumonitis.

DPB is a chronic airway disease that clinically presents with a persistent cough, sputum production, exertional dyspnea, and chronic sinusitis. Radiologically, it is characterized by diffuse small nodules. Historically, it has been associated with chronic inflammatory lesions surrounding the respiratory bronchioles (15). DPB is associated with several diseases, including thymoma (16–26). The precise pathogenesis of DPB remains unclear; however, lymphocyte infiltration and follicular proliferation within the bronchial wall, along with respiratory bronchial thickening, are commonly observed pathological manifestations of DPB (27). Consequently, lymphocytes, particularly T lymphocytes, are believed to significantly influence the pathogenesis of this condition (5, 27). Increased CD8+ T lymphocytes and a decreased CD4+/CD8+ ratio have been observed in the BALFs of patients with DPB, suggesting that active CD8+ T lymphocytes may play a role in the pathogenesis of the disease (6, 27). Furthermore, reduced CD4+ T lymphocytes may contribute to the activation of CD8+ T lymphocytes (28), which may partly explain the development of DPB in this case.

To better understand the association between DPB and thymoma, we conducted a literature search using the terms “diffuse panbronchiolitis” and “thymoma” in the PubMed database for case reports from inception to July 19, 2024. We found 11 case reports involving 12 patients with both DPB and thymomas (16–25). **Table 1** lists the patient characteristics and highlights of these 12 cases, as well as those of the present case. Among the 13 patients with thymoma and DPB considered in this study, 12 were East Asian, accounting for 92.3% of the cases. Seven patients were diagnosed with DPB after the occurrence of thymoma, while six were diagnosed with both conditions simultaneously. All 13 patients underwent surgery, and 4 also received radiation. Gamma globulin levels were measured in 12 cases (12/13), whereas T lymphocyte counts were assessed in only 3 cases (3/13). Unlike the present case, the two patients who exhibited a decline in CD4+ T lymphocytes also presented with Good’s syndrome. Among the patients who survive, 10 received macrolide antibiotic treatment. Among the three patients who deceased, only one did not receive macrolide therapy.

**Table 1:** Summary of reported cases of thymoma with DPB, including the present case.

Reference	Publication Year/Country	Age at diagnosis of thymoma (years)	Age at diagnosis of DPB	Sex	Race	Type of thymoma	Clinical manifestations	Hypogammaglobulinemia	T lymphocyte level	Thymoma treatment	DPB treatment	Outcome (Follow-up period)
(16)	1991/Japan	58	58	F	East Asian	Malignant thymoma invading the pericardium	Productive cough, dyspnea	No	CD4^+^/CD8^+^:1.65	Surgery and irradiation therapy	Erythromycin	Died
(23)	2003/Japan	64	65	F	East Asian	Invasive thymoma	Dyspnea and a productive cough	Yes	CD4^+^/CD8^+^: 0.88	Surgery and irradiation therapy	Erythromycin 0.6 g/d for 6 months	Improvement (6 months)
(21)	2003/China	22	22	F	East Asian	Encapsulated thymoma	Dry cough and fever	No	ND	Surgery	Roxithromycin 0.3 g/d for 3 years	Improvement (3 years)
		54	54	F	East Asian	Encapsulated thymoma	Cough, copious mucopurulent sputum, and dyspnea on exertion	No	ND	Surgery	Roxithromycin 0.3 g/d for 2 years	Improvement (2 years)
(20)	2004/China	38	41	F	East Asian	ND	Cough, expectoration, and dyspnea after exercises	No	CD4^+^/CD8^+^ ratio increased	Surgery	Erythromycin	Improvement
(19)	2010/Indian	ND	50	F	ND	B2-type (WHO classification)	Cough, mucoid sputum and exertional dyspnea	No	ND	Surgery	Azithromycin 0.5 g for 6 months	Improvement (6 months)
(26)	2012/Japan	35	45	F	East Asian	B2-type (WHO classification)	Productive cough, postnasal drip, dyspnea on exertion	Yes	ND	Surgery and chemotherapy	Combination of azithromycin (0.5 g/d) and clarithromycin (0.4 g/d)	Improvement (7 months)
(17)	2014/Japan	43	55	M	East Asian	B3-type (WHO classification)	Wheezing, persistent cough, and dyspnea	No	CD4^+^/CD8^+^: 0.23	Surgery, corticosteroid, and radiation therapy	Clarithromycin 0.4 g/d	Died of progression of DPB and serious respiratory infection at the age of 58 years
(18)	2021/Italy	68	69	M	Caucasian	B1-type (WHO classification)	Exertional dyspnea and productive cough	No	ND	Surgery	Azithromycin 0.5 g/d for 6 months	Improvement (6 months)
(22)	2021/China	63	67	F	East Asian	NA	Recurrent cough and expectoration	Yes	CD4^+^ T lymphocytes were significantly decreased	Surgery	Azithromycin (0.5 g, qod) for 1 year	Improvement (3 years)
(24)	2023/China	53	67	F	East Asian	NA	Recurrent cough, sputum production, and fever	Yes	CD4^+^ T lymphocytes were significantly decreased	Surgery	None	Died due to severe lung infection (After a diagnosis of DPB 2 years later)
(25)	2024/China Taiwan	38	45	F	East Asian	NA	Productive cough, rhinorrhea, anosmia, ear fullness, shortness of breath, and weight loss.	ND	ND	Surgery	Azithromycin (0.5 g, qod) for 1 year	Improved
Present case	China	47	47	M	East Asian	B2-type (WHO classification)	Cough, expectoration, and shortness of breath	No	CD4^+^ T lymphocytes were significantly decreased	Surgery and radiation therapy	Azithromycin 0.25 g/d for 2 years	Improvement (6 months)

DPB, diffuse panbronchiolitis.

NA, not available.

ND, not documented.

A review of the literature and the present case highlights several points that warrant further investigation. First, the role of cellular immune deficiency in the pathogenesis of DPB in patients with thymomas has not been thoroughly explored. Cellular immune deficiency is more prevalent among patients with thymoma than the classical manifestation of Good’s syndrome but has been underestimated (29). Although the mechanisms underlying CD4+ T cell immunodeficiency in patients with thymoma are not well understood, reported cases consistently involve surgical intervention, thymectomy, and radiation (11–13). Thymomas can stimulate lymphocyte proliferation at rates comparable to those in the fetal thymus and, under the influence of thymic epithelium, these lymphocytes differentiate into CD4+ or CD8+ cells, which are subsequently released into the peripheral blood (30). Thus, thymectomy may significantly decrease the peripheral T-cell population, particularly in cases of lymphocyte-rich or hematopoietic thymomas (11, 13). Nakagawara et al. observed a decrease in peripheral blood lymphocytes in a patient following radiation therapy for thymoma and speculated that radiation therapy was the primary cause of CD4+ T lymphocyte defects (11). Wickemeyer et al. reported a patient with thymoma who experienced prolonged CD4+ T lymphocyte deficiency for over 7 years after thymectomy and radiation (12). However, neither study determined whether the cellular immune deficiency was due to the thymoma itself, surgical intervention, or radiation therapy. Among the reported cases and the present case, T-lymphocyte counts were not assessed after thymoma diagnosis, before thymectomy and post-surgery, or before thymectomy and radiation therapy, obscuring the distinction between cellular immune deficiency due to thymoma and subsequent treatments. Based on the findings from this case and previous reports, attention should be paid to assessing cellular immune function in patients with thymoma, both pre-and post-surgery, thymectomy, and radiation therapy. Further research on the relationship between radiation therapy and cellular immunodeficiency in thymomas is required.

Second, macrolide antibiotics play a crucial role in improving the prognosis of patients with thymoma and DPB. Historically, DPB has been considered a nearly fatal disease. However, advances in medical treatment, particularly erythromycin, have significantly improved the 5-year survival rate of patients with DPB, achieving a rate exceeding 90% (14). DPB is now recognized as a condition that can be effectively treated with early diagnosis and intervention. Erythromycin, one of the earliest drugs used for DPB, along with other 14-member and 15-member ring macrolides, has demonstrated definite therapeutic effects. Notably, 16-member ring macrolides have been found to be ineffective for DPB (31). The therapeutic benefits of macrolides for DPB are primarily attributed to their anti-inflammatory and immune-regulatory effects rather than their direct anti-infective properties (5). Erythromycin reduces sputum production by blocking chloride channels in epithelial cells and inhibits the secretion of inflammatory factors and the activity of inflammatory cells. Additionally, erythromycin can inhibit biofilm formation by *Pseudomonas aeruginosa* (32, 33). In this case, chest CT before thymectomy revealed scattered small nodules, suggesting that DPB could have been present before thymectomy. Moreover, following thymectomy and radiotherapy, the patient experienced worsening respiratory symptoms, increased nodules, and tree-in-bud signs on chest CT. Given the significant therapeutic effectiveness of low-dose macrolide antibiotics in managing DPB, early initiation of these antibiotics is likely to confer considerable benefits to patients in similar clinical scenarios before undergoing thymectomy and radiotherapy.

Advances in thymectomy and radiation technologies have significantly reduced the incidence of adverse reactions associated with these treatments (9). Radiation pneumonitis, a detrimental outcome of radiation therapy, was confirmed in this case through the history of radiation treatment, presence of opacities within the irradiated field, exclusion of other pulmonary abnormalities, and rapid resolution of opacities with steroid therapy. The effectiveness of steroid therapy in managing radiation pneumonitis underscores the importance of timely recognition and intervention.

In cases where patients with thymoma exhibit symptoms such as persistent cough, sputum production, and dyspnea, coupled with lung imaging that reveals multiple small nodules and tree-in-bud patterns, it is crucial to consider the potential coexistence of DPB. A thorough patient history, particularly regarding chronic sinusitis, along with pulmonary function tests, can facilitate the diagnosis of DPB. Moreover, evaluating CD4+ T lymphocyte levels is advisable. It is important to note that surgical intervention and radiotherapy may exacerbate DPB; therefore, early identification and treatment with low-dose macrolide antibiotics are essential to enhance patient prognosis.

In conclusion, thymoma and DPB are rare and both generally have a good prognosis when treated promptly. Clinicians should increase their awareness of DPB in patients with thymoma, particularly in those with cellular immunodeficiency and scattered small nodules in imaging. Thymectomy and radiation therapy may contribute to the onset and worsening of cellular immune deficiency and DPB. Therefore, early treatment with macrolides can enhance the prognosis of patients with thymomas and DPB.

## Data Availability

The original contributions presented in the study are included in the article/**Supplementary Material**. Further inquiries can be directed to the corresponding authors.
