# Supplementary material for: Diffuse panbronchiolitis as a rare complication of thymectomy and radiation therapy in a patient with thymoma: a case report
Source: Front Oncol. 2025 Jan 30;15:1496693. doi: 10.3389/fonc.2025.1496693 (PMC11821482; doi:10.3389/fonc.2025.1496693)
Supplement: Supplementary file 1 [file Table1.docx]

Supplementary Material

# Supplementary Table 1 Main laboratory findings of the patient

|  | **Value** | **Reference range** |  | **Value** | **Reference range** |
| --- | --- | --- | --- | --- | --- |
| **Arterial blood gas analysis** | | | **Immunoglobulin panel** | | |
| PH | 7.44 | 7.35-7.45 | IgG (mg/dl) | 12.10 | 6.95-15.15 |
| PaO_2_ (mmHg) | 65 | 75-100 | IgA (mg/dl) | 0.991 | 0.97-3.2 |
| PaCO_2_ (mmHg) | 41 | 35-45 | IgM (mg/dl) | 0.494 | 0.4-1.59 |
| **Blood routine** | | | **Peripheral T lymphocyte absolute count** | | |
| White cell count (×10^9^ cells/L) | 5.38 | 3.5-9.5 | CD3^+^ cell count(cells/ul) | 595 | 690-2540 |
| Neutrophil count (×10^9^ cells/L) | 3.8 | 1.9-7.2 | CD4^+^ cell count (cells/ul) | 253 | 410-1590 |
| Lymphocyte count (×10^9^ cells/L) | 1.0 | 1.1-2.7 | CD8^+^ cell count(cells/ul) | 318 | 190-1140 |
| Hemoglobin (g/L) | 153 | 130-172 | CD4^+^/CD8^+^ ratio | 0.8 | 0.71- 2.78 |
| Platelet count (×10^9^ cells/L) | 223 | 135-350 |  |  |  |
| **Blood chemistry** | | | **Immunological parameters** | | |
| Total protein (g/L) | 74.4 | 60-83 | ANA titer | 1:80 | ＜1:80 |
| Albumin (g/L) | 44.5 | 35-53 | ANA spectrum | Negative | Negative |
| ALT (U/L) | ＜6 | 0-40 | Rheumatoid factors (IU/mL) | ＜ 20 | 0-30 |
| AST (U/L) | 12 | 5-34 | Complement C3 | 1.170 | 0.74-1.4 |
| Urea (mmol/L) | 5.38 | 3-9.2 | Complement C4 | 0.245 | 0.12-0.36 |
| Creatinine (μmol/L) | 58.2 | 59-104 | MPO-ANCA | positive | negative |
| Potassium (mmol/L) | 4.12 | 3.5-5.5 | PR3-ANCA | negative | negative |
| Sodium (mmol/L) | 140 | 136-145 | P-ANCA | negative | negative |
| C-reactive protein (mg/L) | 6.63 | 0-8 | C-ANCA | negative | negative |
| **BALF** | | | **sACE (U/L)** | 26.9206 | 0-52 |
| Smear of bacteria, fungus, and TB | Negative | Negative | **D-dimer (ug/L)** | 224 | 0-252 |
| Culture of bacteria and fungus | Negative | Negative | **T-spot** | Negative | Negative |
| PCR-TB | Negative | Negative | **HIV antibody** | Negative | Negative |
| Macrophages (%) | 3% |  | **Urine analysis** |  |  |
| Lymphocytes (%) | 7% |  | Urinary protein | Negative | Negative |
| Neutrophils (%) | 90% |  | Urine occult blood test | Negative | Negative |
| **Sputum culture of bacterium, and fungus** | Negative | Negative | White cells /HPF | 0.90 | 0.1-2.2 |
| **Sputum smear of TB** | Negative | Negative | Red cells /HPF | 0.80 | 0.1-2.2 |
| **1-3-β- glucan** | ＜37.5 | ＜70 |  |  |  |

PaO_2_，arterial partial pressure of oxygen; PaCO_2_，arterial Partial Pressure of Carbon Dioxide; ALT，alanine aminotransferase; AST, aspartate aminotransferase; IgG, Immunoglobulin G; IgA, Immunoglobulin A ;IgM, Immunoglobulin M; MPO, myeloperoxidase; HIV, human immunodeficiency virus; BALF, bronchoalveolar lavage fluid；ANCA, Anti-neutrophil cytoplasmic antibody; ANA, Antinuclear antibody; TB, tuberculosis; sACE, serum angiotensin-converting enzyme;
